# The doctor’s presence created a safe space - a mixed methods study of students’ learning outcomes from an elective course in palliative medicine

**DOI:** 10.1186/s12909-024-06226-z

**Published:** 2024-11-08

**Authors:** Margrethe Aase Schaufel, Jan Henrik Rosland, Dagny Faksvåg Haugen

**Affiliations:** 1 Department of Clinical Medicine K1, University of Bergen, Bergen, Norway; 2 Department of Thoracic Medicine, Haukeland University Hospital, Bergen, Norway; 3 Regional Centre of Excellence for Palliative Care, Western Norway, Haukeland University Hospital, Bergen, Norway

**Keywords:** Medical education, Palliative medicine, Learning outcomes, Doctor’s role

## Abstract

**Background:**

Competence in palliative medicine is required in clinical practice. Based on a literature review, we developed a two-week elective course in palliative medicine for 5th and 6th year medical students. We wanted to study learning outcomes from the course, especially related to knowledge, confidence, and reflections on the doctor’s role in palliative care.

**Methods:**

A multiple-choice questionnaire (MCQ) assessed knowledge in palliative care pre and post course. The Thanatophobia Scale (TS) and the Self-efficacy in Palliative Care Scale (SEPC) measured confidence in communication with patients close to death and in providing palliative care, respectively. Reflection notes were analysed using Systematic Text Condensation, a cross-case thematic analysis. Lave & Wenger’s theory about situated learning was used to support interpretations.

**Results:**

From 2018 to 2022 we ran four courses for a total of 48 students. Test results improved over the course in all four groups. On average, MCQ scores increased by 22% (range 13–33), TS scores were reduced by 28% (24–32), and SEPC scores increased by 50% (42–64), reflecting increased confidence in dealing with seriously ill and dying patients and their relatives. The participants prepared reflection notes describing their main impressions and take-home messages from the course, focusing specifically on the role of the doctor. They described the doctor’s role linked to an overarching task of creating a sense of security for patients and relatives. Through the course, and especially through talking to patients and relatives and being part of the interprofessional team, the participants learned how this sense of security was built by gaining competence in the following domains: 1) Patient-centred communication about the disease, expected trajectory, and needs, establishing common ground and support; 2) Being the medical expert in symptom relief and decision-making, providing guidance and reassurance in difficult situations; 3) Professionalism rooted in a holistic and relational approach; and 4) Being a good team player, aware of their function and limitations.

**Conclusions:**

A two-week student-selected course in palliative medicine improved knowledge and skills and increased confidence in providing palliative care. The comprehensive understanding of the doctor’s role obtained in this course may also be relevant to other clinical specialties.

**Trial registration:**

Not applicable (no clinical trial).

**Supplementary Information:**

The online version contains supplementary material available at 10.1186/s12909-024-06226-z.

## Background

Palliative medicine provides clinical leadership, care, and support to prevent and relieve suffering for people with life-limiting and life-threatening illness [1]. When the disease can no longer be cured or controlled, focus is on alleviating distressing symptoms and providing comprehensive care for the patient and relatives. Only a limited number of doctors work full time in palliative medicine, but most residents and consultants encounter seriously ill and dying patients in their work. Thus, knowledge and skills in palliative medicine are emphasized and required in several specialties, such as internal medicine and medical and surgical oncology [2–4]. Basic training in palliative medicine has been established in medical schools, but with considerable variation across universities and countries [5, 6].

The World Health Organization recommends that “palliative care should be integrated as a routine element of all Undergraduate Medical Education” [7]. The European Association for Palliative Care (EAPC) published in 2013 recommendations for the scope and content of palliative medicine education for undergraduate students [8]. The minimum requirement was a total of 40 h during medical school. The different recommended subject areas are shown in Table 1. More recently, the EAPC recommendations were reviewed and revised in the international EDUPALL project, and the revised recommendations translated into an updated curriculum document [9].

**Table 1:** EAPC recommendations for the palliative medicine subject during medical school [8]

Recommended topics (indicative proportion of tuition indicated in brackets) 1. Basics of Palliative Care (5%) 2. Pain and symptom management (50%) 3. Psychosocial and spiritual aspects (20%) 4. Ethical and legal issues (5%) 5. Communication (15%) 6. Teamwork and self-reflection (5%)	

Palliative medicine was implemented in the study plan for medical students at the University of Bergen, Norway, already in 2006, with a total of 37 h allocated through the six years of training. In 2015, a new study plan was introduced, placing the subject “Pain and Palliative Care” in the 5th year. The new plan also introduced elective periods in which students would have the opportunity to delve into certain subjects. One month each of the last three years of medical school, regular teaching would be replaced by elective courses. We wanted to offer a student-selected course in palliative medicine, in addition to the basic education offered to all students as part of the general program and assess learning outcomes from the elective course.

## Methods

### Aim

We set up a study to investigate learning outcomes from the elective course in palliative medicine at the University of Bergen, focusing particularly on the doctor’s role in palliative care.

### Design

**Fig. 1:**
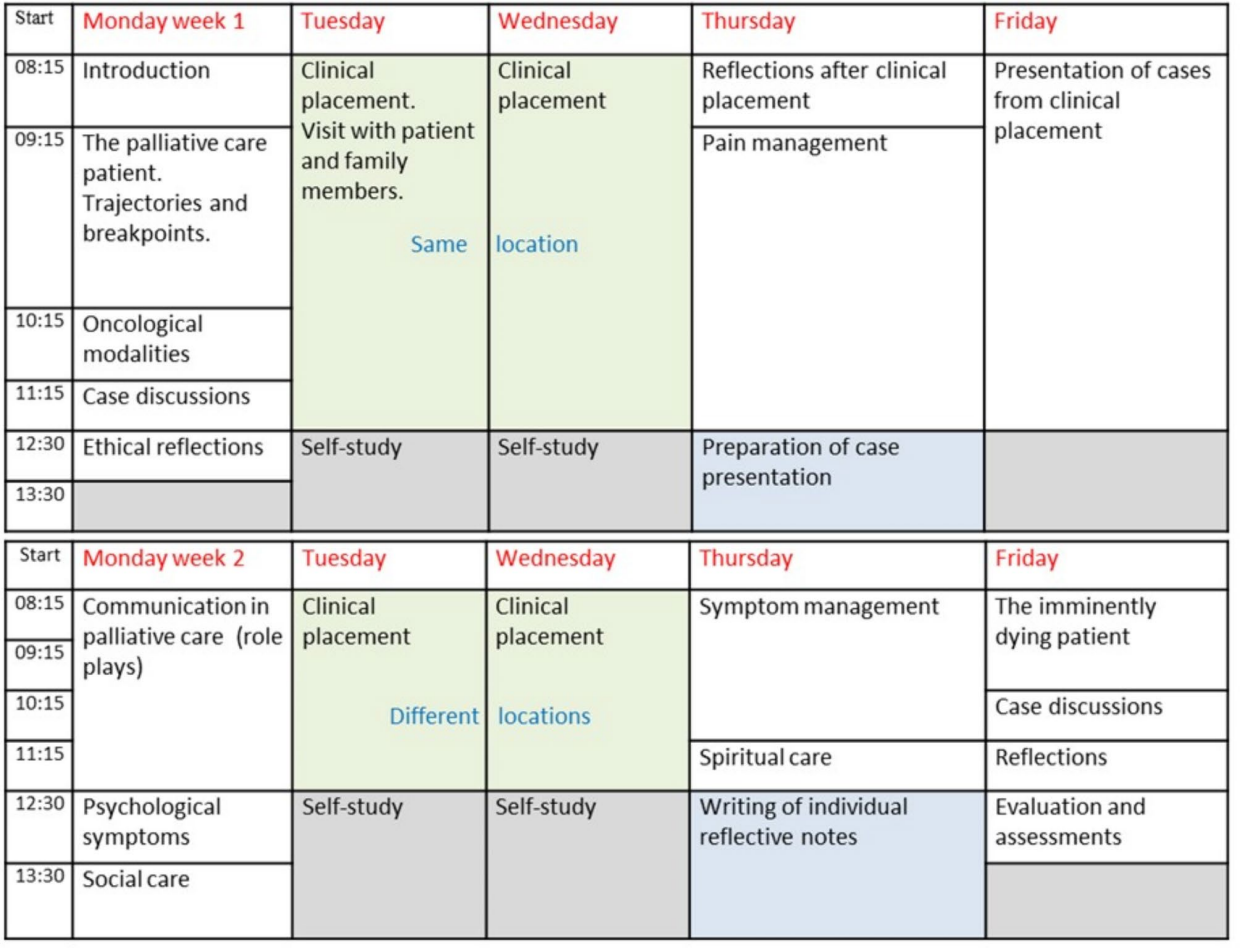
Timetable for the elective course in palliative medicine

Based on a literature review, a two-week elective course in palliative medicine for 5th and 6th year medical students was developed in 2016–2017. The course was designed for a maximum of 15 students and addressed the following themes: palliative care philosophy and principles, pain and symptom management, psychosocial and spiritual/existential challenges, ethical reflection, communication, and care for the dying patient. It included four days of clinical placement at different services providing palliative care. The students presented a patient case from one of their placements and handed in two written assignments and a reflective note during the two weeks. Classroom teaching was interactive and case-based, with no standard lectures but a lot of group work and discussions. Communication skills training was based on the VitalTalk approach [10, 11]. The timetable for the course is shown in Fig. 1.

A multiple-choice questionnaire (MCQ) was developed for this study (by two of the authors) to assess knowledge in palliative care pre and post course (see supplementary file). It contained 18 questions about pain and pain management, symptom management, prognostication, communication, and the general palliative care approach. These core competencies and the reasoning behind the correct answers were based on common clinical cases, EAPC guidelines [12] and National guidelines for palliative care in Norway [13]. The Thanatophobia scale (TS) and Self-efficacy in Palliative Care Scale (SEPC) measured confidence in communication with patients close to death and in providing palliative care, respectively [14, 15]. The students handed in a reflection note in which they were invited to elaborate on their most important learning outcomes, impressions, and thoughts, especially regarding the doctor’s tasks and role when providing palliative care. They were encouraged to reply to the following questions:

- What did you learn from the course?
- What do you take along for future use?
- Has anything made a special impression on you?
- What do you think about the doctor’s tasks and role in relation to palliative care patients?

### Ethics approval and consent to participate

All participants gave informed consent to participate in the study. The need for ethics approval was deemed unnecessary according to the Norwegian Health Research Act [16] and the regulations of the Norwegian Agency for Shared Services in Education and Research (Sikt) [17], due to the nature of the research and the full anonymity of the respondents.

### Participants

Since 2018, we have run four courses for a total of 48 students. The sample size comprised all these students, since everybody consented to participate. The two first courses had students from both the 5th and 6th year of study, as this was the minimum requirement for elective courses. We realized, however, that it was problematic to have students on the course who had not passed the general “Pain and Palliative Care” subject in the 5th year, and this was accepted by the program leaders. From 2021, only 6th year students participated.

### Analysis

A mixed methods design was used to assess learning outcomes. Descriptive statistics were used to analyse questionnaire data. For the MCQ, each correct answer gave one point. Results are given as mean total score per course (maximum obtainable score 18 points). The SEPC scale contains eight items on communication, eight items on patient management, and seven items on multidisciplinary teamworking, 23 items in total. Each item is scored on a visual analog scale (VAS), anchored on one end “very anxious” and the other “very confident”. When analysing the scores, the VAS was transformed to a 0–15 numerical rating scale (NRS) (0 = very anxious; 15 = very confident). Due to missing data (some students not scoring all items), results are given as average mean score per item. The Thanatophobia Scale measures feelings associated with thanatophobia, or fear of death, and addresses the individual’s feeling of discomfort in dealing with dying patients [18]. This scale has seven items scored on a seven-point ordinal scale anchored on one end by “strongly disagree” and by “strongly agree” on the other. Scores are calculated by summing the selected points for all seven items. The higher score, the greater discomfort in dealing with dying patients. Results are given as mean total score. Reflection notes from 47 students (1 missing) were analysed using Systematic Text Condensation, a cross-case thematic analysis proceeding through four steps [19]: (1) reading all the material to obtain an overall impression; (2) identifying units of meaning, representing different aspects of the participants’ learning outcomes, and coding for these; (3) condensing and abstracting the meaning within each of the coded groups; and (4) summarizing the contents of each code group to generalized descriptions and concepts reflecting the most important learning outcomes reported by the participants. A decision trail documented the choices during the analytical process [20]. Lave & Wenger’s theory about situated learning was used to support interpretations [21]. This theory describes learning as an interactional activity between individuals in a community of practice within cultural and institutional structures and has been used when analysing doctors’ learning processes in end-of-life care [22]. The analysis was, however, conducted in an editing and not theory-driven style [23].

## Results

**Fig. 2:**
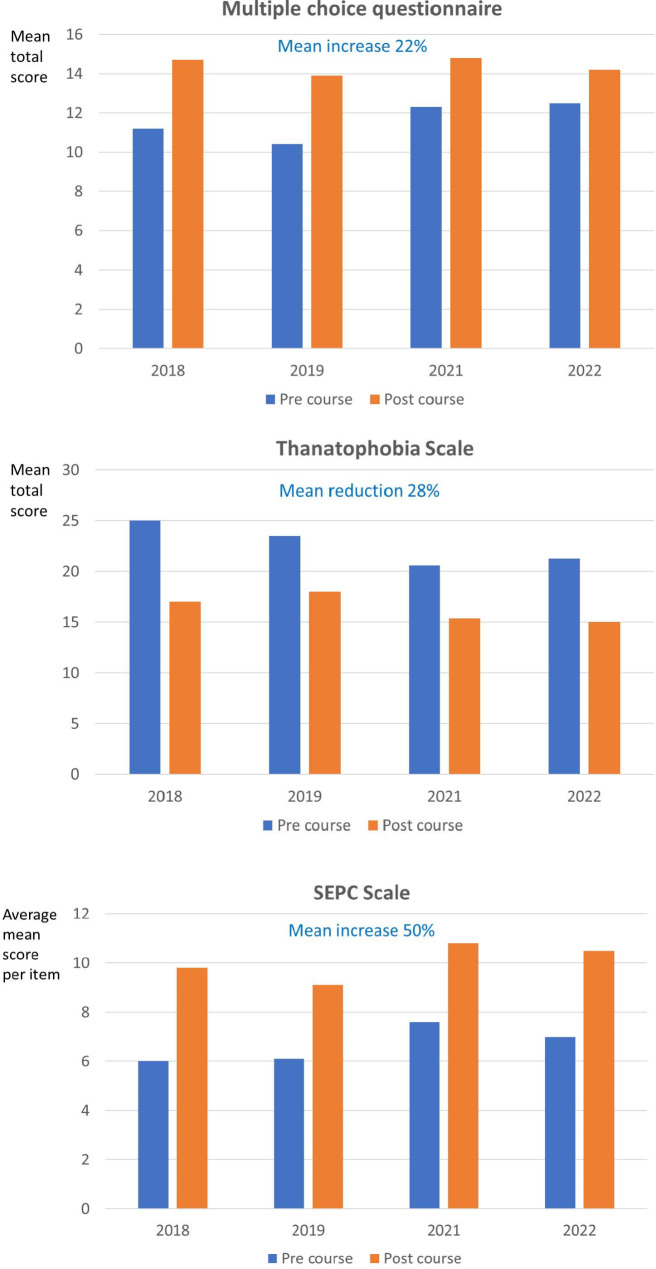
Pre and post measurements of knowledge (multiple choice questionnaire), confidence in communication with patients close to death (Thanatophobia Scale) and self-efficacy in palliative care (SEPC scale)

The test results improved from the start of the course to the end of the course for all groups (Fig. 2). Scores for the MCQ increased on average by 22% (range 13–33). TS scores were on average reduced by 28% (24–32) and SEPC scores increased by 50% (42–64), as a sign of increased confidence when dealing with seriously ill and dying patients and their relatives.

The participants described the doctor’s role linked to an overarching task of creating safety for the patients and their relatives in the patients’ last period of life. This safety was built by gaining competence in the following domains: (1) Patient-centred communication about the disease, expected trajectory, and needs, establishing common ground and support; (2) Being the medical expert in symptom relief and decision-making, providing guidance and reassurance in difficult situations; (3) Professionalism rooted in a holistic and relational approach; and (4) Being a good team player, aware of their function and limitations (Table 2). Below, we elaborate our findings with quotations from the reflection notes.

**Table 2:** Experienced learning outcomes about the doctor’s role in palliative care

Creating safety for the patients and their relatives			
Patient-centred communication	Being the medical expert	Holistic and relational professionalism	Being a good team player
•Establish relationship and mutual understanding • Impart customised information • Communicate about different dimensions of life	• Expert in symptom relief and disease management • Expertise in decision-making • Provide guidance and reassurance	• From preserving life to safeguarding quality of life • Holistic focus • Emphasize personal presence	• The benefit of working in teams • The doctor’s coordinating tasks

### Patient-centred communication

#### Establish relationship and mutual understanding

The participants experienced how the doctor’s tasks entailed creating a relationship in which the patients could feel secure and trying to arrive at a common understanding of their situation. This required a good therapeutic alliance right from the start, and they mentioned that effort should be made to achieve this, especially in the first conversations. They stated that training to bring out all the patients’ troubles and decipher the most urgent task gave them skills that were not only important in palliative medicine, but also in other specialties. Since several patients did not dare bring up what bothered them, they saw how important it was that the doctor created a safe environment. The students discovered how the doctor’s role involved daring to “open the door to difficult conversations ajar and see if you are invited in”, often earlier in the process than they first thought was necessary. The importance of being able to listen was emphasized, as the patients’ thoughts and needs differed so much. Some students were nervous asking the patient personal questions but described how they had been challenged during the course to stand against what they feared, and had learned that openness, shown with consideration and caution, was beneficial to the patient.

*“I would like to point out what I think are the most important roles a doctor has when dealing with seriously ill and palliative care patients. I believe that it is extremely important to listen and try to understand the patient’s needs and wishes. You must try to create a good relationship with safety and enough time. At the same time*, *it is the doctor’s task to convey difficult but important information to the patient and relatives and take the initiative to talk about topics such as death and end-of-life issues*, *if the patient does not do this him/herself. Still*, *it is important not to push them too far if the patients express that they do not want to talk about this now.” (Student 8)*.

#### Impart customised information

The students comprehended how a doctor’s work in palliative care rests on good communication skills. An important focus, and at the same time a challenge, was to inform the patient about prognosis, treatment options, and uncertainty. This required honesty, vulnerability, and time. They saw that it was necessary for the doctor to provide customized information and at the right time. This comprised exploring patients’ understanding of their disease and information preferences. Some were surprised by how frankly many patients wanted to talk about their illness. Participants learnt that it could be a mistake to be reticent, because it could rob patients and relatives from important choices, the opportunity to come to terms with the situation and say goodbye. The patients’ reservations regarding information had to be respected, while the doctor was responsible for providing appropriate information about and preparing the patient for the time ahead. Balancing hope and realism was described as an important and interesting learning activity during the course. The students reflected on how their own personality played a role, and that they might want to hold back a little too much for the sake of the patient because they were afraid of causing discomfort. They had seen information giving that was exemplary, but also doctors whom they thought imparted overwhelming details. Role-plays were helpful and enhanced understanding of the patient’s perspective:

*“The balancing act between not taking away people’s last hope*, *but at the same time not giving them false hope*, *was extremely interesting to take part in.” (Student 46)*.

#### Communication about different dimensions of life

The participants presented how they had learned to communicate about patients’ diverse needs, both through theoretical teaching and practice. They had experienced how patients’ concerns often included practical challenges, psychological problems, or spiritual issues. When the doctor was able to acknowledge these matters, they had seen how patients felt respected and cared for. The students acknowledged that the doctor had to answer questions of existential character in a sensitive way, as it was important for the patients’ well-being and quality of life. They recognized the importance of having reflected as a doctor on existential aspects of life. The spiritual problems patients may face before death were not something the medical students had thought through before the course. Some mentioned how crucial it was for patients to find peace with themselves before their life ended, and that neither society nor medical school prepared them to face this. The doctor’s responsibility for taking the patient’s social situation into account, detecting conflicts, and uncovering financial problems was also perceived. They had noticed that the doctor needed knowledge about the various services for challenges other than purely medical ones. Participants comprehended how important it was to take a family’s background into account, and how taking the time to talk to the next of kin could be necessary to get in a position to talk about what was important to all stakeholders.

*“In society*, *death is something terribly frightening*, *in medical school death is something terribly clinical. None of the arenas have prepared me for the existential questions that often arise. While death can be gloomy*, *sad and painful*, *this course has also shown me that death can be something beautiful*, *close and unifying. The strength that is brought forth*, *and the care that is shown in families*, *are truly incredible.” (Student 15)*.

*“I have also realized how important the patient’s relatives are. Before this course*, *I probably thought a little less nuanced about the patient’s next of kin. I have to admit that I hadn’t really thought much about the importance of relatives’ influence on the illness trajectory. I guess I sat with the idea that it is the patient who must be the priority here. After all*, *it is the patient who is ill. I still think that*, *but with more perspectives*, *a little wiser and a little more nuanced.” (Student 47)*.

### Being the medical expert in symptom relief and decision-making

#### Expert in symptom relief and disease management

The doctor’s role was primarily understood as having the medical responsibility for the treatment. Knowledge of how to alleviate the various symptoms the seriously ill or dying patient experiences was described as fundamental. Furthermore, the participants had learned how doctors had to be aware of complications that could arise from the disease or medication. The students acknowledged how much it meant to the patients that the doctor was confident when explaining and managing palliative care interventions according to what was most important for the patient. They expressed learning about how the doctor had to be able to map complex symptoms and distress, constantly assessing new complaints or lack of treatment response. It could be a challenging job, as you constantly had to weigh the benefits of treatment against side effects, and then often had to be a bit ahead of the curve and treat prophylactically. The students perceived how important it was to plan well in advance and think a couple of steps ahead when caring for palliative care patients. They emphasized how this could prevent suffering, avoid misunderstandings, and create an opportunity for reconciliation for the patient and family.

*“The doctor must have the ability to anticipate what may come around the next bend and be able to make a plan together with the patient for various possible challenges and outcomes. That’s how good palliative care is provided.” (Student 43)*.

#### Expertise in decision-making

The participants described how the doctor’s role in palliative medicine implied being the one who makes treatment decisions. They recognized that their job was to determine what treatment the patient would benefit from, based on their professional knowledge and experience. This had to be done together with the patient, relatives, and other healthcare professionals, and at the same time they saw that it could be difficult for patients to assess whether the treatment was useful or not in a palliative phase. From the conversations they participated in, students discovered how important it was that the doctor must clearly bear responsibility for decisions related to issues such as treatment limitations. This could be difficult, especially when patients wanted to be kept alive as long as possible and receive all available treatment. The participants mentioned that it had been very useful to see and hear case reports from experienced doctors about how challenging it could be to break bad news, terminate life-prolonging treatment, make do-not-resuscitate orders, and handle several other issues. They acknowledged how new cancer treatments made it more difficult to make decisions about when to say that enough is enough, and that the patient and the doctor could assess this differently.

*“The doctor’s role in palliative medicine has to do with being the one who makes treatment decisions. These must be taken in consultation with the patient*, *relatives and nurses who know the patient*, *but it is the doctor who must ensure that the patient receives comprehensive treatment and care.” (Student 23)*.

#### Provide guidance and reassurance

Being the medical expert, the students saw that the doctor was in a special position when it came to creating security. This could be done in many ways, both directly in conversation with the patient and their relatives and more indirectly by looking ahead and taking the initiative to map and plan the way ahead. The participants described how the doctor in palliative medicine should be somebody constant and safe for the patient during a time that could be unpredictable and frightening. The doctor could reassure the patient and their relatives, provide support, and even ensure patients and relatives a peaceful and meaningful time at the end of life. From clinical practice during the course, participants learned that just being there could be even more powerful than prescribing the most alleviating medications. They argued that if you were uncertain in such situations, it would quickly be disclosed and in this way destroy the safety you were trying to provide. The students therefore wondered whether they needed to be extra confident academically to work in this field, and hoped they could be a safe haven for their patients in their future work facing hopelessness, fear, anxiety and shock. Having observed how colleagues managed this, they wanted to create the same atmosphere. They had learnt that in some situations doctors could not do much more than being present, and that it was important to dare do so.

*“I think that security is the most important thing you as a doctor can give a patient in a palliative phase. Confidence that we will do our best to alleviate symptoms that arise*, *and confidence that we are available and will be able to make decisions for them and with them*, *and make changes if needed and the patient himself is not able to do so. Not least also a reassurance that we are there if the patient needs a conversation partner.” (Student 29)*.

*“I felt that the doctor’s presence created a safe space for the family.” (Student 41)*.

### Professionalism rooted in a holistic and relational approach

#### From preserving life to safeguarding quality of life

Understanding and acceptance of the core principles of palliative medicine were described as the first and most important step towards becoming a good and reflective palliative care provider. The students perceived that palliative medicine was essentially different from other specialties. When patients could not recover, the focus was on having as good quality of life as possible the time they had left. Participants suggested that it could be challenging to “take off one’s curative coat” yet underlined the importance of taking a step aside from the underlying disease and focus on symptom relief. They had observed how staff in palliative care units managed to make this shift, but how some hospital departments emphasized differently upon discharge, such as providing extensive and unrealistic nutritional plans at the end of life. These observations made them reflect on their upcoming role, in which they wanted to see the vital things for the patient. Participants had become aware that this often did not imply making the patient live as long as possible, but as well as possible, causing a minimum of discomfort and harm. Based on patient encounters throughout the course, they gained a deeper understanding of patient preferences, such as spending as much time as possible with friends and family without nausea, pain, and fatigue. The students realised that disease-directed interventions were not always the best choices when aiming for quality of life. Rather, they saw that sometimes doctors had to turn down their inclination for medical interventions and instead focus on what could bring out patients’ hope and create a good day.

*“Perhaps the most difficult thing to deal with in the beginning was the approach one must have in palliative medicine: The focus is on palliative care and a dignified life*, *and not necessarily on treating the disease at all costs. This was very different from what we have experienced so far in medical and surgical wards.” (Student 1)*.

#### Holistic focus

Some students emphasized that the most important thing they had learned was to see the whole human being and not just a patient with a disease. Showing empathy, being a fellow human being and respecting the patient were fundamental. They pointed out that the course had highlighted positive things about the patient’s life, and how to make their entire situation a little better. Participants no longer regarded palliative care as sad or depressing, but rewarding, because patients were treated in a completely different way than on most wards. Especially through clinical placements, they noted that the focus was completely different from previous teaching. The focus was not primarily on the underlying disease, but the complex person they were caring for. The students especially valued getting in close contact with the mindset and practice that facilitated a dignified end of life. Some even wrote that this had been crucial to their development into a caring and compassionate doctor.

*“Palliative care is not just about pain relief*, *other symptom relief or treatment of disease. It is about the whole person in his complex wholeness. It’s about death. It is about how we can help a person*, *at his most vulnerable*, *to meet the end of his/her life journey in a dignified and respectful way*, *free from pain*, *free from suffering and hopefully free from fear. It’s about communication and humanity. It’s about understanding and acceptance. It’s all about seeing the individual and really listening and understanding what is important to the individual*, *and how we in the best possible way can help and arrange for them to be able to enjoy the time they have left to the best of their ability and as hassle-free as possible.” (Student 47)*.

#### A professionalism emphasizing personal presence

The opportunity to get close to patients and relatives was described as rewarding. This made it easier to understand which dreams were taken away, which relationships were complicated, which ambitions were fading away, and what expectations for the future needed to be reshuffled. At the same time, these experiences were also difficult. One student described how a seriously ill patient of the same age made a strong impression, both the pain that made the patient scream, the dismal prognosis, and the loneliness the student sensed. In this encounter, the student felt powerless not being able to help. At the same time, insight emerged to avoid thinking that one should have all the answers, but rather decide to tolerate taking part in the patient’s pain. Proximity could require putting aside the doctor-patient relationship a little and just be a fellow human being. This called for time and quiet to be established, but the participants had seen how surprisingly little time was needed. Some noticed that when they wore a doctor’s coat and showed their presence, patients often confided in them and disclosed their vulnerability. It was perceived as a privilege to be in this role. At the same time, the students emphasized that the doctor also had to protect his or her private sphere and obtain an ability to accept situations as they are in order to cope with the loss and suffering of others.

*“The good thing about palliative medicine is that you get on a deeper level with the patients and their families. There are many beautiful and fragile moments*, *and the doctor is truly privileged to participate in these moments.” (Student 21)*.

*“If I were to pick one specific thing to take away from this course*, *I think it would have to be respect*, *humility*, *and curiosity. (….) It is a lesson that gains more and deeper layers the more one learns and the more one experiences*, *and I am constantly amazed at how many doors these three principles open both for oneself and for others*, *if only one is able to live by them.” (Student 47)*.

### Being a good team player, aware of their function and limitations

#### The benefits of working in teams

The students acknowledged that palliative care was all about interdisciplinary work. Being part of a team including nurses, psychologists, social workers, physiotherapists, priests, and others, made them realise that they were less limited by their own lack of experience and abilities. In collaboration with the rest of the team, the patient and relatives, an individualized, comprehensive care plan could be established. The communication they observed between nurses and doctors conveyed that they were equal partners in providing the best possible care. Some participants had experienced a doctor tell them that if anyone in the team disagreed with the care plan, they should speak up and a new discussion be held. The students described doctors who showed great understanding of their role as helpers and members of a team, not aiming at being the main characters. The good cooperation in the interprofessional team appeared exemplary. Sometimes they saw that the doctor had a minor role, and that, for example, a social worker was more important to the patient than medical expertise.

*“Palliative care issues often require interdisciplinarity*, *where the doctor is one of many important pieces. There are many palliative care issues where the doctor would never have reached the goal alone*, *but where he/she must work together with other professional groups.” (Student 40)*.

#### The doctor’s coordinating tasks

Nevertheless, they observed that the doctor often had a leading role in the interdisciplinary team when identifying the patients’ needs. They recognized the doctor as a coordinator with the goal of alleviating total suffering, and that this was manageable in collaboration with the interdisciplinary environment they had around them. The students appreciated that it was wise to frequently assess whether a colleague from another profession might be better qualified to solve a task than themselves. In their encounter with the complex palliative care patient, they saw how many considerations had to be taken into account and combined with different disciplines and agencies to ensure a good and dignified end of life. Some students reported having been surprised when a consultant asked them for feedback or advice after a conversation with a patient. They had not experienced such behaviour and attitudes in other medical specialties, and outlined how this created a good working environment and also increased patient safety. The students had noticed that the way the doctor communicated things to the collaborating parties affected how for instance homecare nurses could cope with the situation, which in turn could affect both physical and mental symptoms for the patient. They comprehended what doctors could do to facilitate a good illness trajectory.

*“Even though all members of the team are equally valuable*, *it is often the doctor who has the greatest overview and thus the «leader» role. It is therefore important to have the ability to receive information from everyone who has to do with the patient*, *make deliberation together with the team*, *use of the right professions*, *and choose the treatments that are best for the patient.” (Student 17)*.

## Discussion

This study demonstrates how a two-week course enabled students to reach a broad spectrum of palliative care learning goals. In particular, a profound understanding of the doctor’s role was described. Below, we discuss the implications of our findings, as well as strengths and limitations.

### Communication as key competence

Our findings display how a two-week course enabled medical students to understand the fundamental importance of communication skills. Even though communication courses are integrated into most undergraduate programs worldwide, attitudes towards such training may be unfavourable [24]. The participants in our study underlined the powerful learning experience of witnessing how customised information and the ability to listen were key competencies when tailoring appropriate interventions. This aligns with previous research demonstrating how education in palliative medicine may increase communication skills [25]. The students also revealed a profound understanding of challenges that could arise in the doctor-patient-dialogue and were exposed to key skills such as balancing honesty with hope [11]. They also experienced how “non-medical” issues could be essential to discuss with the patient and relatives. Instead of being critical towards this as part of the doctor’s work, they rather conveyed attitudes of acknowledgement and appreciation. We believe this is a particularly important learning outcome, since leading experts in clinical communication highlight that “skills trump tools, but attitudes trump skills” [26]. Participating in medical practice where both communication skills and favourable attitudes towards patient-centred care are exhibited may promote students’ professional qualities [27].

### Learning complex decision-making integrated in relational professionalism

One of the main reported learning outcomes regarding the multifaceted role of the doctor, was being responsible for difficult treatment decisions. Ethical dilemmas linked to overtreatment and priority setting were addressed, and participants comprehended how doctors tried to respect autonomy while at the same time avoiding harmful therapies. This balance may be difficult to achieve, even for experienced clinicians [28]. Previous research has shown how end-of-life care training can assist interns to adjust decisions according to the patient’s life story [22]. Our informants experienced how decision-making was situated in a “safe space” created by the doctor, which is among patients’ main requests when discussing end-of-life issues [29]. The students learned how their mere presence as medical experts could install tremendous support, and at the same time be a rewarding and meaningful experience. Throughout the course, role models displayed doctor-patient-relationships emphasizing partnership and empathy [30]. This demonstrated how to accompany persons with life-threatening illness and ameliorating suffering as state-of-the-art in medical practice [31]. The students outlined how these experiences of situated learning inspired them in their professional development.

### Palliative care training develops skills required across specialties

Our findings are in line with previous research showing that palliative care training offers insight into a medical field which in several ways differs profoundly from other specialties [32]. However, palliative care education may also offer competence and attitudes relevant to several other clinical disciplines [33]. Interprofessional collaboration and holistic care are crucial when caring for patients with multimorbidity and life-threatening diseases. Planning ahead of expected events in an illness trajectory, as the students reported learning during the course, is of huge importance in clinical practice and should be more emphasized in healthcare professional education [34]. The social nature of learning and medicine as a community of practice may help educators identify sustainable and stimulating learning arenas [35]. Palliative care training focused on experiential learning may foster skills required to meet challenges in modern medicine across specialties.

### Strengths and limitations

Data from four courses, and using both qualitative and quantitative measures, enabled a thorough investigation of our objective. Even though the course has been run and evaluated by one medical school only, we believe our findings may be relevant in other settings. Since communication and openness about end-of-life issues and needs are highly influenced by cultural values and traditions, some of our findings may not be transferable to medical education in settings differing substantially from Western societies.

A challenge related to reflection notes and self-assessment is the chance of students overestimating their achievements and using phrases from learning material. The participants were also likely to be more interested in and motivated to learn palliative medicine than many fellow students due to their signing up for this student-selected course. However, sharing vulnerable aspects of their learning process, such as difficulties in communication with patients, made it likely that they imparted experiences and thoughts in honest terms, ensuring internal validity. This study was not designed to evaluate long-term effects of undergraduate training in palliative medicine, or observation of students’ skills.

Our course has been continued after 2022. The course requires a good deal of coordination and planning, especially concerning the clinical placements in various palliative care services across our region. Communication skills training with role plays is done in small groups, requiring several facilitators. For most of the classroom teaching, two faculty are present. This means that the course is rather resource demanding. Limiting the course to 6th year students was a clear advantage, ensuring that all participants had basic palliative care knowledge.

## Conclusion

This two-week student-selected component in palliative medicine improved knowledge and skills and increased confidence in providing palliative care. Our findings suggest that medical students by participating in this course obtained a comprehensive understanding of the doctor’s role in palliative medicine, which also may be relevant to other clinical specialties. This competence encompassed patient-centred communication, expertise in symptom relief and decision-making, a professionalism rooted in a holistic and relational approach, and being a good team player. Practical learning such as patient interactions or role play was most valued by the students and has been emphasized when continuing the course.

## Electronic supplementary material

Below is the link to the electronic supplementary material.

Supplementary Material 1

## Data Availability

The quantitative dataset used and analysed during the current study is available from the corresponding author on reasonable request. The reflection notes used for qualitative analysis are not available in full text due to our participants’ confidentiality, but a decision trail is available on request.
