# Supplementary material for: The doctor’s presence created a safe space - a mixed methods study of students’ learning outcomes from an elective course in palliative medicine
Source: BMC Med Educ. 2024 Nov 8;24:1282. doi: 10.1186/s12909-024-06226-z (PMC11549761; doi:10.1186/s12909-024-06226-z)
Supplement: Supplementary file 1 — Supplementary Material 1 [file 12909_2024_6226_MOESM1_ESM.docx]

**MCQ – ELMED 308 –** correct answers in **bold** text

**A** A 70-year-old man with lung cancer and bone metastases has experienced increasing pain in his right hip. The pain started 6 weeks ago, and he has had good effect from 4-6 tab. codeine-paracetamol combination (Paralgin forte) daily. But for the past week, he has been taking 8 tab. codeine-paracetamol combination daily without much help. The pain is constant, aching, localized to the hip, without radiance.

1. Increasing pain in this patient is probably due to:

1. Development of tolerance
2. Increasing depression
3. Dependence
4. **Disease progression**

2. The probable pain mechanism:

1. Neuropathic pain
2. **Somatic pain**
3. Visceral pain
4. Vascular pain

3. When will you expect the maximum effect of 2 tab. codeine-paracetamol combination?

1. **After 30-45 min**
2. After 1-1.5 hours
3. After 2-2.5 hours
4. After 3-3.5 hours

4. The best way to give him better pain relief from now on:

1. Oral morphine as needed
2. Oral methadone as needed
3. Oral paracetamol and oral morphine as needed
4. **Oral paracetamol and oral morphine prolonged-release as per dosing schedule**

5. Additional medication that should be tried **first** if not sufficient relief from the above regimen:

1. Sarotex (amitriptyline)
2. Neurontin (gabapentin)
3. **Ibux (ibuprofen)**
4. Lyrica (pregabalin)

6. How will you prevent constipation in this patient:

1. Not necessary, the risk of this is small as he is used to opioids
2. Moventig tablets (naloxegol)
3. **Movicol powder stirred into water (polyethylene glycol)**
4. Dulcolax tablets (bisacodyl)

**B** A 35-year-old woman who has been treated for locally advanced breast cancer, including the use of paclitaxel, has had increasing, burning pain under both feet for the past 2 months. The pain keeps her awake at night. She is numb when walking and unable to walk barefoot on a rough sandy beach. She has been prescribed codeine-paracetamol combination by her GP, but does not think it helps.

7. How would you classify these pains?

1. **Neuropathic pain**
2. Visceral pain
3. Somatic pain
4. Referred pain

8. What would you offer her to help with her pain?

1. Switching to Dolcontin (morphine prolonged-release) and Paracet (paracetamol)
2. Add Naproxen (naproxen)
3. **Adding Sarotex at night (amitriptyline)**
4. Switching to Fentanyl patch and Paracet

**C** A 67-year-old woman with advanced pancreatic cancer comes to the outpatient clinic with her husband. Over the past month, she has lost her appetite completely, and lost significant weight. She has been lying on the couch and does not have energy to do anything. She has just been told that she cannot receive any more chemotherapy.

Her spouse stops you outside the room, pulls you aside, and whispers: "If you have more bad news, tell me, not her. She can't take any more!"

9. How should you respond to what her spouse is saying?

1. Suggest that the two of you meet afterwards and review the situation
2. Tell her spouse that for us, patient autonomy is most important and that we need to be honest with her
3. Ask her spouse to explain in more detail what I can say to her and what I should not say
4. **Ask her spouse to explain a little more about his concerns, preferably together with the patient**

10. The best prognostic factor for survival in advanced cancer:

1. **Functional status**
2. Metastatic spread
3. Serum albumin
4. Pain intensity

11. The patient asks you, "How much time do you think I have left?" You check out that both she and her spouse want you to say something about the prognosis. You answer the following:

1. On average, patients with your type of disease live 6-9 months
2. Only God knows how long you have left
3. **I believe that you have a short time left, most likely weeks to a few months**
4. It is completely impossible to say how much time you have left

12. She asks, "Is there anything I can do to improve my appetite?"
Which of the following drugs has a documented effect on appetite in patients with advanced cancer:

1. Haldol (haloperidol)
2. **Dexamethasone (dexamethasone)**
3. Sobril (oxazepam)
4. Docetaxel (docetaxel)

13. A few weeks later, she is hospitalized due to increasing dyspnea and further reduced general condition. A chest CT reveals significant blurring on the right side consistent with pleural effusion and/or infiltrate. What do you propose as a measure?

1. Morphine to relieve dyspnea
2. **Ultrasound to assess pleural effusion**
3. Referral for radiotherapy
4. Starting antibiotic treatment

**D** You are visited at the doctor's office by a 54-year-old woman with breast cancer, who has recently been prescribed morphine tablets at the Cancer Department. She is nauseous and has trouble keeping the tablets down. She thinks she can't tolerate morphine.

14. Which claim about morphine and nausea is true:

1. **The main cause of nausea with morphine is stimulation of the vagus nerve and delayed emptying of the stomach**
2. Nausea caused by morphine is usually accompanied by itching
3. Nausea from morphine is an early sign of morphine allergy
4. Nausea from morphine rarely goes away by itself

15. What treatment do you suggest to alleviate her nausea?

1. Explain the reason to her and wait until it passes
2. **Write a prescription for Afipran (metoclopramide)**
3. Write a prescription for Zofran (ondansetron)
4. Write a prescription for Marzine (cyclizine)

16. The patient wants to switch to Fentanyl patch as she has heard from a friend that this causes less nausea. How long does it take for the patch to have a stable effect?

1. 2-6 hours
2. 7-12 hours
3. **13-24 hours**
4. 24-36 hours

**E** A 76-year-old widow living alone with long-standing COPD is admitted to a short-term stay in a nursing home after having had 6 emergency hospitalizations in the past year due to acute dyspnea with low O2 saturation and confusion. She has each time recovered by use of intermittent BIPAP and antibiotic treatment.

17. When will you as a nursing home doctor plan an advance care planning conversation?

1. It is not necessary since she will only have a short-term stay
2. As soon as she shows new signs of worsening of her COPD
3. **At the first doctor's appointment after arrival at the nursing home**
4. After you have clarified it with her family

**F** A 70-year-old woman with a tendency to fall and emaciation has been diagnosed with ALS. You are the patient's GP and receive a phone call from her spouse saying that he is exhausted and needs help.

18. What do you do?

1. Call the homecare service and ask them to visit
2. Apply for a short-term stay in a nursing home
3. **Arrange a collaborative meeting in the patient’s home together with the homecare service to map the situation**
4. Ask the spouse and patient to come to your office to take blood tests and get an overview of the situation
